# Antivenom Evaluation by Electrophysiological Analysis

**DOI:** 10.3390/toxins9030074

**Published:** 2017-02-23

**Authors:** Rita Restano-Cassulini, Walter Garcia, Jorge F. Paniagua-Solís, Lourival D. Possani

**Affiliations:** 1 Instituto de Biotecnologia, National Autonomous University of México, Av. Univeresidad 2001, Col. Chamilpa, Cuernavaca 62210, Morelos, Mexico; 2 Instituto Bioclón S.A. de C.V., Calle Miguel Laurent 427, Del. Benito Juarez, Ciudad de México 03100, Mexico; wgarcia@silanes.com.mx; 3 Laboratorios Silanes, S.A. de C.V., Amores 1304, Colonia del Valle, Ciudad de México 03100, Mexico; jpaniagua@laboratoriosilanes.es

**Keywords:** antivenom, horse immunoglobulin, human ion channels scorpion venoms, sodium channel, patch-clamp, electrophysiology

## Abstract

Scorpion stings on humans are medically relevant because they may contain toxins that specifically target ion channels. During antivenom production, pharmaceutical companies must use a large number of experimental animals to ensure the antivenom’s efficacy according to pharmacopeia methods. Here we present an electrophysiological alternative for the evaluation of horse antivenoms produced against two species of Moroccan scorpions: *Buthus mardochei* and *Androctonus mauretanicus*. Human sodium and potassium channels and acetylcholine nicotinic receptors were analyzed by standard patch-clamp techniques. The results showed that the antivenom is capable of reversing ion current disruption caused by the venom application. We propose the use of this in vitro technique for antivenom evaluation as an alternative to using a large number of live animals.

## 1. Introduction

Scorpion stings may be potentially harmful to humans. Chippaux and Goyffon reported that globally there are over one million cases of scorpion stings every year, of which about 350,000 occur in countries situated in the North African region, leading to an estimated 630 deadly cases [1]. Worldwide there are over 2200 different species of scorpions, comprising 208 genera distributed in 20 families [2,3], for which the fatal incidents usually occur with scorpions belonging to the Buthidae family. Scorpion venoms are rich sources of different biologically active components, used by these arachnids to ensure their survival—either defending themselves from predators or allowing them to capture their prey. A major characteristic of the venoms from scorpions of the Buthidae family is the presence of low molecular weight peptides [4,5] that bind to ion-channels in the envenomated animal (mammals, and arthropods such as crustaceans and insects), causing impairment of the proper function of their excitable cells in muscle and nerve tissues [6]. In the case of humans, stings from dangerous scorpions may cause death if not treated promptly [7,8].

The only effective proven treatment for scorpion stings are various formats of antibodies, principally obtained from horses, and processed before using as F(ab’)_2_ (see review by [9]). According to protocols approved by the World Health Organization, the efficacy of the different types of antivenoms must be confirmed by injecting experimental animals (usually mice) with different doses of venom from a given species of scorpion mixed with the antibodies generated after a prolonged period of immunization [9]. During this process, many thousands of animals are submitted to these challenging experiments, and this procedure has been criticized by organizations promoting more humane treatment of animals. For this reason, there is a great interest in finding new protocols that can be used safely to guarantee the efficacy of the generated antivenoms. Another weakness of the current protocol is that venom target in mice may present subtle differences to that in humans [10]. It has recently been convincingly demonstrated that neutralizing the effect of the Na^+^ channel-specific toxins from the venom of dangerous scorpions is sufficient to ensure survival of an envenomated animal [11]. These toxins have been characterized and shown to affect channel function in two different ways: one is defined as alpha-effect, which delays the sodium channel inactivation process [12]; and the other is defined as beta-effect, which induces the opening of sodium channels at more negative potentials [13,14]. Both types of sodium channel-specific toxins and the presence of potassium channel-blocking peptides were found in the venom of these scorpion species. The physiological effects of these venom components produce an abnormal massive depolarization of the target cells causing impairment of their proper function.

In this communication, we describe the use of various sub-types of human Na^+^-channels—heterologously expressed in cell culture—as a qualitative test to validate the neutralizing property of horse antivenom produced by the Instituto Bioclon S.A. de C.V. in Mexico. The electrophysiological behavior of these ion-channels of human origin was monitored by the application of soluble venom from two important scorpions of North Africa: *Androctonus mauretanicus* (*Am*) and *Buthus mardochei* (*Bm*). As described below, the modification of the function of the channels is obvious when applying the soluble venom and its effect is prevented by co-application of the prepared antivenom. To the best of our knowledge, this is the first time that a systematic assay has been performed using different heterologously-expressed human ion channels to confirm the efficacy of a scorpion antivenom, and could form the basis for novel protocols that drastically reduce the number of living animals required for experimental validation of the anti-toxic effect of scorpion antivenoms.

## 2. Results

### 2.1. *Androctonus mauretanicus* and *Buthus mardochei* Venoms Contain Sodium Channel Toxins

**Figure 1:**
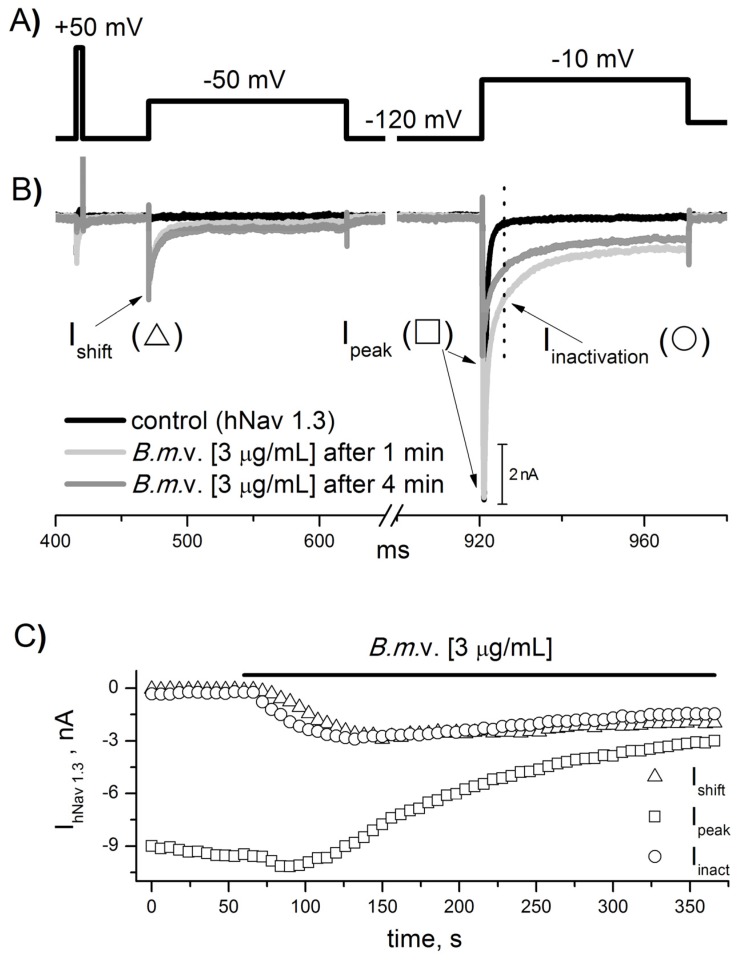
Characterization of venom effects on sodium channels. Sodium currents produced by hNav 1.3 sodium channels were elicited by using the stimulation protocol as described in (**A**). This stimulus was applied every 6 s, and the current values were graphed as a function of time, as shown in (**C**). In control conditions, no channels opened at the sub-threshold potential, whereas all channels opened at the full-activation potential ((**B**), black trace). Application of 3 µg/mL *Buthus mardochei* (*Bm*) venom (*Bm*v) induced current at the sub-threshold potential (I_shift_) and slowed the current inactivation process (I_inactivation_). After one-minute venom application, the total current measured at the full-activation potential slightly increased (I_peak_), but after more prolonged application, the I_peak_ started to decrease (respectively, light gray and dark gray lines in (**B**)). The time-dependence of the venom effect can be seen in panel (**C**), where the black bar corresponds to venom application.

The main objective of this study was the determination of the efficacy (at the molecular level) of the antivenom (av) produced by Instituto Bioclon against the *Androctonus mauretanicus* (*Am*) and *Buthus mardochei* (*Bm*) venoms. The venom of scorpion species dangerous to mammals (including humans) contains toxins that act upon sodium channels that are the principle components responsible for the envenomation symptoms. For this reason, it was decided to first test the effects of the *Am* and *Bm* soluble venoms (*Am*v and *Bm*v) on seven subtypes of human sodium channels expressed in HEK and CHO cells (hNav 1.1–1.7). Venoms were applied during the stimulation protocol described in Figure 1A, where sodium current was first elicited at a sub-threshold potential (−50 mV, or −60 and −70 mV respectively for hNav 1.4 and hNav 1.5) preceded by 5 ms at 50 mV pre-pulse, aimed to prime the channels and then at a full-current activation pulse (−10 mV or −20 mV for hNav 1.5). This protocol was applied every 6 s, and the venom’s effects were plotted against time (Figure 1A–D).

We found that the venom of both species changed the voltage-gated dependence of the sodium currents in a similar way, but with different potencies depending on the type of the venom and the channel sub-type. An example of the venom effect is depicted in Figure 1, where *Bm* venom was applied to the hNav 1.3 sodium channel. After exposure to 3 µg/mL of the venom, the channels started to open at more negative potential (I_shift_, Figure 1B,C), probably due to the effect of toxins of the beta type; at the same time, channels suffered a delay in the inactivation process (I_inact_, Figure 1B,C), probably due to the toxins of the alpha type. In most cases, the peak current (I_peak_) initially increased and then decreased (Figure 1C). The progressive loss of current also reflects the effects of alpha and beta toxin that are described to increase or reduce the total current. We noticed that the delay of inactivation was in general predominant over the shift of activation, indicating that in the *Am* and *Bm* venoms, the effect of the alpha toxins is more prevalent than that of the beta toxins.

A set of experiments similar to that described in Figure 1 was performed for each subtype of sodium channel, applying the *Bm* and the *Am* venoms at concentrations near the previously calculated LD_50_ for *Bm* and *Am* venoms that are, respectively, 10 µg and 5 µg for 20 g mouse body weight (we assumed that 20 g mouse is equivalent to a volume of 1–1.5 mL ). Currents I_shift_, I_peak_, and I_inactivation_ were measured in control conditions and after venom application (3–5 min or until current value appears stable).

### 2.2. NA Scorpion Antivenom Neutralizes the *Androctonus mauretanicus* Venom Effect on Sodium Channels

**Figure 2:**
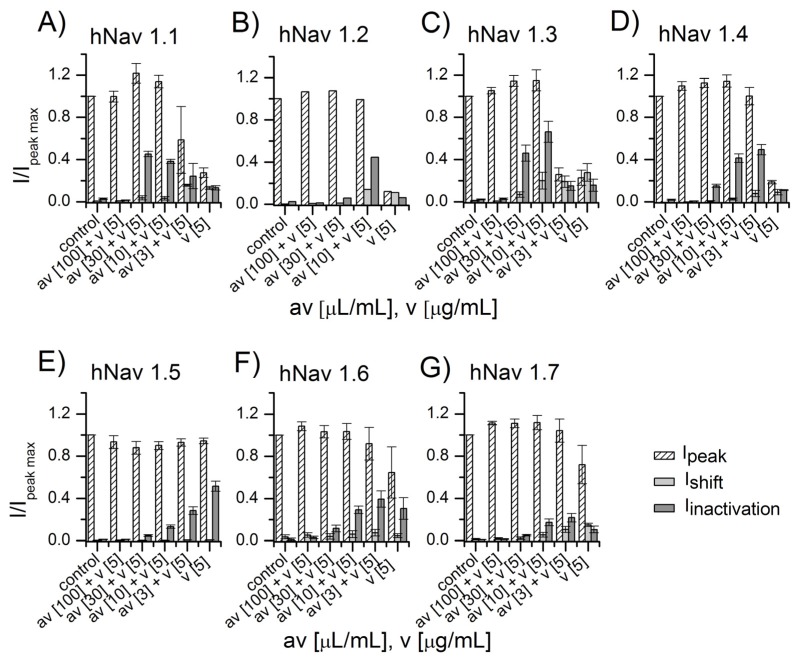
NA scorpion antivenom protection against *Androctonus mauretanicus* (*Am*) venom (*Am*v) in different sodium channel sub-types. Current values of I_shift_, I_peak_, and I_inactivation_ recorded in control are reported and compared with the values of currents recorded after application of *Am* venom along with different antivenom concentrations. Antivenom at 100 µL/mL completely protects against *Am* venom effects upon all sodium channels sub-types (panels **A**–**G**). In hNav 1.2, hNav 1.5, hNav 1.6, and hNav 1.7, antivenom was also protective at 30 µL/mL (panels **B**,**E**–**G**).

When *Am* soluble venom was applied to the sodium channels of the sub-types hNav 1.1-hNav 1.7, current measured at sub-threshold potential (I_shift_) increased, current at full-activation potential (I_peak_) increased and then decreased, and current measured after complete inactivation (I_inactivation_) increased (Figure 2A–G). Venom was then applied along with different NA scorpion antivenom concentrations corresponding to 3, 10, 30, and 100 µL dissolved in 1 mL of extracellular solution. When venom was applied in the presence of 100 µL antivenom, the recorded currents were the same as in control conditions. This means that NA at this concentration was able to completely neutralize the venom effects in all sodium channels assayed (Figure 2A–G).

The mixture of 30 µL/mL of antivenom plus venom still showed full protection for the channel sub-types hNav 1.2, hNav 1.5, hNav 1.6, and hNav 1.7, but only partial protection for hNav 1.1, hNav 1.3, and hNav 1.4. The two latter channel sub-types appeared to be most sensitive to the venom. As shown in Figure 2, the antivenom acts in a dose-dependent manner: at lower concentrations (i.e., 10 and 3 µL/mL), the venom effect is still evident—albeit reduced—compared to its effect in the absence of antivenom.

### 2.3. NA Scorpion Antivenom Neutralizes the *Buthus mardochei* Venom Effect on Sodium Channels

**Figure 3:**
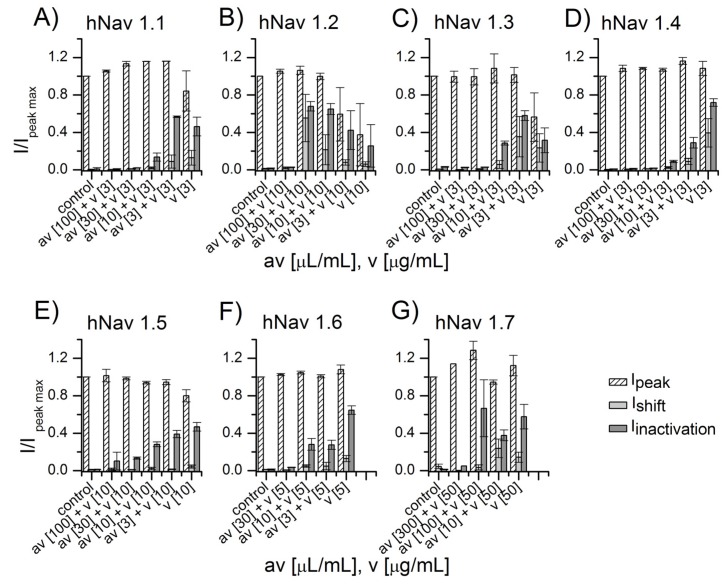
NA scorpion antivenom protection against *Bm* venom in different sodium channel sub-types. Current values of I_shift_, I_peak_, and I_inactivation_ recorded in control are reported and compared with the values of currents recorded after application of *Bm* venom along with different antivenom concentrations. Antivenom at 100 µL/mL concentration completely protects six sodium channel sub-types against *Am* venom (panels **A**–**F**). For hNav 1.7 channels, 50 µg/mL of venom was used. In this case, the minimum antivenom concentration capable of completely neutralizing this amount of venom was 300 µL/mL (**G**).

Experiments similar to those performed with *Am* venom were replicated with *Bm* venom. In this case, the venom was applied at concentration near to the LD_50_, but with slight differences depending on the channel sensitivity. For instance, we used 50 µg/mL of venom on the hNav 1.7 channel, as it proved relatively insensitive to the venom. As before, *Bm* venom was applied alone, or pooled together with increasing concentration of antivenom. Additionally, as before, we found that antivenom completely neutralized the venom’s effect upon all sodium channels sub-types evaluated here, in a dose-dependent manner (Figure 3).

### 2.4. NA Scorpion Antivenom Neutralizes the Am and Bm Venom Effects on Potassium Channels

**Figure 4:**
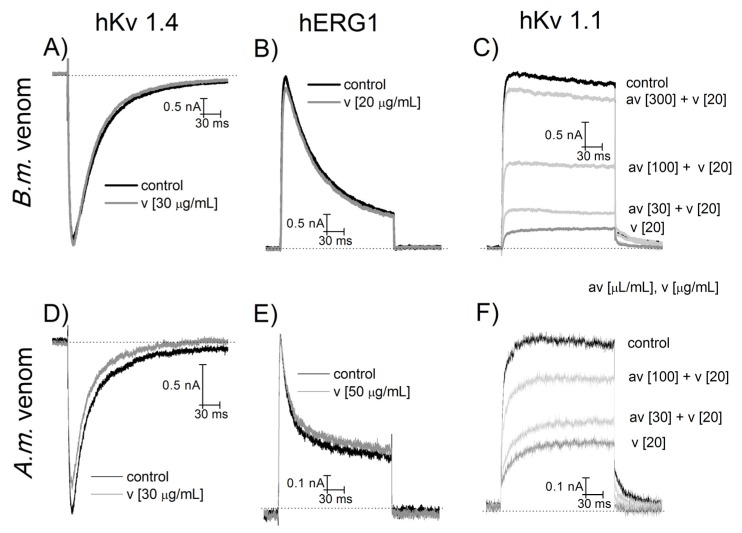
NA scorpion antivenom protection against *Am* and *Bm* venom in potassium channels. Potassium channels of the type hERG, hKv 1.1 and hKv1.4 were recorded in control (black traces in **A**–**F**) and after application of *Bm* (**A**–**C**) and *Am* (**D**–**F**) venoms (dark gray traces). Both venoms reduce hKv 1.1 current. Antivenom, applied at different concentrations, prevents the blocking effect of both venoms in a dose-dependent manner (light grey traces in **C** and **F**).

Scorpion venom of species dangerous to mammals contain toxins that modify the sodium currents, and are responsible for the severe symptoms observed under intoxication. However, these venoms also contain toxins that block the function of potassium channels. For this reason, we investigated whether *Am* or *Bm* venoms contain toxins that act upon human *Ether*-*à*-*go*-*go*-related-gene channels (hERG) and two additional human voltage-gated potassium channels (hKv 1.1 and hKv 1.4). Both venoms did not modify hERG and hKv 1.4 channel parameters at concentrations up to 50 µg/mL, whereas hKv 1.1 currents were reduced when applying each venom at a concentration of 30 µg/mL (Figure 4). As previously, the antivenom was applied at different concentrations in conjunction with venom; also as observed previously, antivenom prevented the hKv 1.1 blockage produced by the *Am* and *Bm* venoms.

### 2.5. Am and Bm Venoms Do Not Modify Normal Functioning of the Acetylcholine Nicotinic Receptor

**Figure 5:**
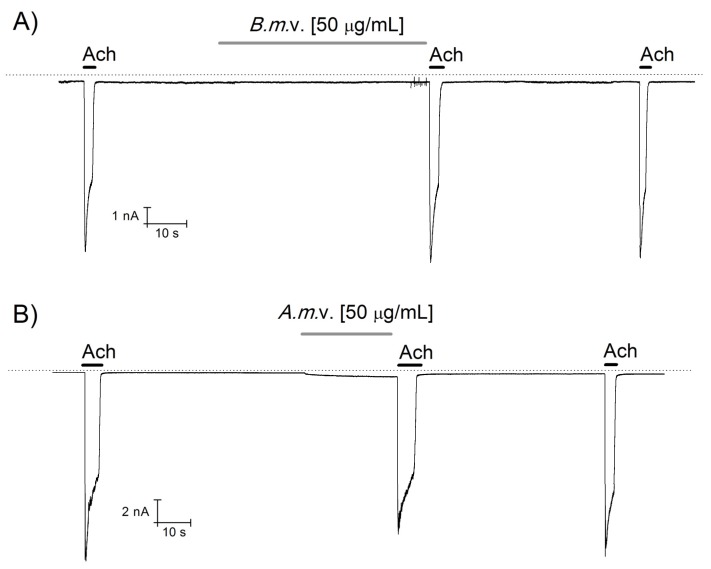
*Am* and *Bm* venoms activity on acetylcholine nicotinic receptor. Currents conducted through nicotinic receptor were elicited by pulse of 10 µM acetylcholine (Ach, black line in **A** and **B**). After application of 50 µg/mL of both *Am* or *Bm* venoms (grey line in **A** and **B**), the current was not significantly modified.

Since scorpion stings induce neuro-muscular alterations, we explored the activity of *Am* and *Bm* venoms on acetylcholine nicotinic receptors expressed in TE671 rhabdomyosarcoma cells in tissue culture. Currents were elicited by applying 10 µM acetylcholine (Ach) in 3–5 s pulses before and after 30–60 s perfusion with 50 µg/mL of *Am* or *Bm* venoms (Figure 5). In these conditions, the venoms did not modify TE671 nicotinic currents. Thus, it was not necessary to further apply the antivenom.

## 3. Discussion

In this communication, the NA scorpion antivenom was produced by the Instituto Bioclon S.A. de C.V. and evaluated for its neutralization efficacy at the molecular level. This antivenom was prepared by hyper-immunizations of horses with venom from two different species of Moroccan scorpions (*Bm* and *Am*), using similar procedures approved for production of “Alacramyn”, another antivenom produced by the same company and capable of protecting against the toxic effects of the scorpions of the genus *Centruroides* of Mexico [15,16]. Animals use venom to capture their prey or to defend themselves from predators. Among the molecular mechanisms of venom activity, the impairment of proper cell communication—mainly between muscle and nerve tissue or nerve terminals and secretory glands—is best-defined [17,18]. Various types of receptors, including ion channels, are the main targets of the deleterious components found in animal venoms, and their functional alterations induced by the venom are well characterized and defined by electrophysiological experiments [19,20,21,22]. Major advances in the field of toxin-binding studies have been facilitated by the availability of many cloned genes coding for human ion-channels. These advances suggest the possibility of using specifically-raised horse immunoglobulins for neutralization of the perturbations caused by the venom components (toxins) on the membrane receptors (ion channels). The protective activity of the same antivenom analyzed in the present study was previously assessed in the chick biventer cervicis preparation, and the results obtained on the neuromuscular system were compared with lethal activity neutralization in mice [17]. In this case, the findings were achieved using a tissue preparation derived from another animal model (different from mice).

For the experiments described in the present work, seven different sub-types of Na^+^-channels of human origin were used (hNav1.1 to 1.7). Moreover, three sub-types of potassium channel (hKv1.1, hERG, and hKv 1.4) and the nicotinic acetyl-choline receptor were tested for function in normal conditions and in the presence of various concentrations of venoms from *Am* and *Bm*. The antivenom was able to protect the functioning of the sodium ion channels in a dose-dependent manner, as shown in Figure 2 and Figure 3. Antivenom efficacy was also proved when using hKv1.1 potassium channels (Figure 4). Since hERG, hKv1.4, and the nicotinic acetylcholine receptor were not affected by the application of both venoms, it was not necessary to confirm protection of the antivenom against the functioning of these membrane-bound receptors (Figure 5).

Two important points emerged from the experiments reported in this communication. The first is that by using in vitro cell culture systems, the deleterious effects of scorpion venom components can be verified without employing a great number of living animals (usually experimental mice), which in current protocols are challenged in vivo for validation of the efficacy of antivenoms. The second most significant finding is that the ion-channels used for heterologous expression are of human origin. Due to the small, but potentially significant differences that exist between ion-channels of experimental animals and humans [10], the protection seen on receptors of human origins in cell culture are more representative of real-life scenarios in which humans suffer a scorpion sting. Whilst a quantitative comparative analysis of both in vivo and in vitro methodology is still necessary, we are nonetheless confident that protocols derived from these experiments could present a valid alternative method for approval by the regulatory authorities to replace the currently employed in vivo assays that use a wide range of live animals.

## 4. Materials and Methods

### 4.1. Cell Culture

All cells used in this communication were cultured in high glucose DMEM (Dulbecco Modified Eagle Medium) (Sigma, Toluca, Mexico) supplemented with 10% Fetal Bovine Serum (ByProductos, Guadalajara, Mexico) and maintained at 37 °C in a humidified incubator. Antibiotic G418 at 400 µg/mL concentration was added to the medium for HEK cells expressing sodium channels and CHO cells expressing hERG potassium channel. Cells expressing hNavs sodium channels and plasmid for hERG channel expression were a kind gift from Professor Enzo Wanke from University of Milano-Bicocca. Nicotinic acetylcholine receptor was endogenously expressed from TE671 human rhabdomyosarcoma cells [23].

### 4.2. Solutions

For sodium currents, external solution contained (in mM): 130 NaCl, 5 KCl, 2 CaCl_2_, 2 MgCl_2_·6H_2_O, 10 HEPES, 5 glucose, pH 7.3 adjusted with NaOH. Intracellular solution contained (in mM): 105 CsF, 27 CsCl, 5 NaCl, 2 MgCl_2_, 10 EGTA, 10 HEPES, pH 7.3 adjusted with CsOH. For acetylcholine nicotinic currents and potassium currents of the type hKv 1.1 and hKv 1.4, external solution was the same as for sodium current experiments, and the internal solution contained (in mM): 130 K-aspartate, 10 NaCl, 1.3 CaCl_2_, 2 MgCl_2_·6H_2_O, 10 HEPES, 10 EGTA, pH 7.3 adjusted with NaOH. For hERG potassium current experiments, extracellular solution contained (in mM): 95 NaCl, 40 KCl, 2 CaCl_2_, 2 MgCl_2_·6H_2_O, 10 HEPES, 5 glucose, pH 7.3 adjusted with NaOH. Intracellular solution was the same as for acetylcholine nicotinic current experiments.

### 4.3. Source of Venom and Antivenom

Lyophilized venoms were obtained from Latoxan, Portes lès Valence, France. To prepare the stock solution, venoms were dissolved in distilled water and centrifuged at 14,000× *g*, and the supernatant was stored at −20 °C.

The lyophilized antivenom used in this communication was called NA scorpion antivenom. It was produced by Instituto Bioclon S.A. de C.V., Mexico City, Mexico. Stock solution was prepared by dissolving the content of one ampoule in 5 mL of extracellular solution and maintaining it at 4 °C until its use. The final work concentration was expressed in µL (of the stock solution)/mL.

### 4.4. Records

The MultiClamp 700B amplifier along with the analogue–digital converter Digidata 1440A and software pClamp10 (Molecular Devices, Sunnyvale, CA, USA) were used during current recordings. Sodium currents were elicited by means the of stimulation protocol described in Figure 1A: first, channels were primed with 5 ms pre-pulse at 50 mV and after 50 ms at −120 mV, membrane potential was depolarized at sub-threshold potential (−50 mV or −60 mV and −70 mV, respectively, for hNav 1.4 and hNav 1.5); after a repolarization step at −120 mV, membrane was further depolarized at a full activation potential (−10 mV or -20 mV for hNav 1.5). The stimulation was repeated every 6 s. Potassium currents of the type hKv 1.1 and hKv 1.4 were elicited by a step depolarization at 60 mV for 200 ms, followed by a step at −50 mV for 200 ms. Pulses were applied every 6 s. hERG currents were elicited as tail currents by means of a depolarized step at 60 mV for 500 ms followed by a repolarization step at −120 mV for 500 ms. Pulses were applied every 1.1 s. Nicotinic currents were elicited by applying a pulse of 10 µM Ach for 3–5 s. Ach pulses were repeated every 50–60 s until currents appeared stable. Venoms were applied for 30–60 s or until remaining currents stabilized.

Data were analyzed with the software Clampfit10 (Molecular Devices, Sunnyvale, CA, USA) and Origin7 (OriginLab, Northampton, MA, USA).
